# Ventromedial prefrontal cortex activity during extinction recall suggests successful extinction learning via mental imagery

**DOI:** 10.1093/scan/nsag031

**Published:** 2026-05-07

**Authors:** Andrew L Lyons, Xinrui Jiang, Thomas Rawliuk, Lauryn Burleigh, Scarlett Horner, Laurent Grégoire, Steven G Greening

**Affiliations:** Brain and Cognitive Sciences, Department of Psychology, University of Manitoba, Winnipeg, Manitoba, R3T 2N2, Canada; Cognitive and Brain Sciences, Department of Psychology, Louisiana State University, Baton Rouge, Louisiana, 70803, United States; Brain and Cognitive Sciences, Department of Psychology, University of Manitoba, Winnipeg, Manitoba, R3T 2N2, Canada; Cognitive and Brain Sciences, Department of Psychology, Louisiana State University, Baton Rouge, Louisiana, 70803, United States; Brain and Cognitive Sciences, Department of Psychology, University of Manitoba, Winnipeg, Manitoba, R3T 2N2, Canada; Cognitive and Brain Sciences, Department of Psychology, Louisiana State University, Baton Rouge, Louisiana, 70803, United States; Department of Psychological and Brain Sciences, Texas A&M, College Station, Texas, 77843, United States; Brain and Cognitive Sciences, Department of Psychology, University of Manitoba, Winnipeg, Manitoba, R3T 2N2, Canada; Cognitive and Brain Sciences, Department of Psychology, Louisiana State University, Baton Rouge, Louisiana, 70803, United States; Centre on Aging, University of Manitoba, Winnipeg, Manitoba, R3T 2N2, Canada

**Keywords:** fear extinction, extinction recall, mental imagery, vmPFC, fear

## Abstract

Mental imagery is an important element of psychological therapies for fear-related disorders. The present study evaluated the efficacy of imagery extinction using a within-subject design allowing for the comparison of a conditioned stimulus extinguished via mental imagery (CS+Ei) to an unextinguished conditioned stimulus (CS+U) during extinction recall. Twenty-eight human participants underwent differential fear conditioning during functional magnetic resonance imagining. Successful acquisition of differential fear conditioning was confirmed by self-reported fear, skin conductance response (SCR), and activation the anterior insula and dorsal anterior cingulate cortex. Next, participants imagined the CS+Ei without mild shock and the CS− during the imagery extinction learning phase, in which fear transfer was confirmed with self-reported fear and SCR. Lastly, all stimuli were again visually presented in the visual extinction recall phase. Significantly greater ventromedial prefrontal cortex (vmPFC) activity was observed for the CS+Ei compared to the CS+U, suggestive of successful extinction recall following imagery extinction. Moreover, a vmPFC seed-based psychophysiological interaction analysis indicated that unlike the CS+Ei, the CS+U was positively connected with bilateral amygdala during extinction recall. The present study highlights dissociable vmPFC involvement during extinction recall when viewing the CS+Ei following fear extinction using mental imagery as compared to the unextinguished CS+.

## Introduction

Mental imagery is the perceptual-like experience devoid of sensory input from external stimuli (Lewis *et al*. 2013). Imagery is an important component to both the symptomology of anxiety disorders (e.g. intrusive flashbacks; Hirsch and Holmes 2007) and its clinical treatments (e.g. imaginal exposure; Holmes and Mathews 2010). Many existing studies that have investigated the connection between imagery and emotion have employed fear conditioning paradigms (Agren *et al*. 2017, Grégoire and Greening 2019, Greening *et al*. 2022, Lyons *et al*. 2024). Such a paradigm repeatedly pairs an initially neutral stimulus (CS) with an aversive stimulus (US; e.g. mild shock). The pairing of the US and CS results in a CS+ that elicits fear-associated responses, which is commonly compared to a second stimulus (CS−) that was never paired with the US. The learned fear responses can then be extinguished with repeated exposure to the CS+ without the accompanying US pairing (Milad *et al*. 2007, Pace-Schott *et al*. 2013, Fullana *et al*. 2018). Such extinction learning studies, using within-subject designs, fear condition participants to two CS+, whereby one will be extinguished (CS+E) and the other remains unextinguished (CS+U). Participants are then reintroduced to both CS+, facilitating an examination of the CS+E to the CS+U during extinction recall. Recently, similar extinction results have been achieved by imagining the CS+ devoid of the US (CS+Ei), rather than physically perceiving the CS+ (Agren *et al*. 2017, Reddan *et al*. 2018, Jiang and Greening 2021). Related studies have also demonstrated the effectiveness of mental imagery in fear extinction using reinstatement (Hoppe *et al*. 2022) and reconsolidation procedures (Grégoire and Greening 2019). However, no research to date has compared a CS+Ei to a CS+U during extinction recall.

Previous functional magnetic resonance imaging (fMRI) studies have reported various brain regions associated with fear conditioning and/or extinction learning and recall, including the amygdala, anterior insula cortex (AIC), dorsal anterior cingulate cortex (dACC), and ventral medial prefrontal cortex (vmPFC; Labar *et al*. 1998, Phelps *et al*. 2004, Milad *et al*. 2007, Delgado *et al*. 2008). In two meta-analyses, Fullana and colleagues examined the neurological signatures of fear conditioning (Fullana *et al*. 2016) and fear extinction (Fullana *et al*. 2018). Unlike many independent studies on fear conditioning, Fullana *et al*. (2016) did not find robust involvement of the amygdala, rather, they found consistent activation of AIC and dACC and deactivation of vmPFC. Similarly, in the fear extinction meta-analysis, Fullana *et al*. (2018) again reported no significant amygdala involvement. Instead, they also observed greater activity in the AIC and dACC during extinction learning. Importantly, during extinction recall, greater vmPFC activity was observed for the CS+E versus CS+U (Fullana *et al*. 2018), consistent with the role of the vmPFC in extinction recall and fear attenuation (Phelps *et al*. 2004, Milad *et al*. 2007). Related studies of instructed and vicarious fear extinction have similarly supported greater vmPFC activity during extinction recall (Golkar *et al*. 2016, Javanbakht *et al*. 2021). Regarding the lack of amygdala findings, Wen *et al*. (2022) demonstrated recently that amygdala activity is involved during early trials but rapidly habituates. Supporting these results, a recent mega-analysis of 2199 participants corroborated the significant CS+ > CS− activation in the right amygdala during only the early portion of fear conditioning trials (Radua *et al*. 2025).

To date, no literature exists regarding the impact of imagined extinction learning on the neural correlates of extinction recall. However, previous research evaluating the neurological underpinnings of extinction learning via mental imagery has been conducted. Using a between-subjects design, Reddan *et al*. (2018) demonstrated that imagining a CS+ tone was as effective as listening to the CS+, as reflected in attenuated SCR and increased vmPFC activity following extinction learning.

The current study tested whether repeated imagery of a CS+ could replicate perceptual extinction studies comparing the CS+E to CS+U during extinction recall (Milad *et al*. 2007, Pace-Schott *et al*. 2015). Utilizing a within-subject design, participants were differentially fear conditioned to two CS+ and one CS−. Immediately after this Acquisition phase, participants repeatedly imagined only one of the two CS+ (CS+Ei) along with the CS−, absent the US. Finally, participants visually perceived both CS+ (CS+Ei, CS+U) and the CS− again in an extinction recall phase. We hypothesized that fear responses, as measured by self-report and skin conductance response (SCR) would be reduced for the CS+Ei as compared to the CS+U. Further, if imagery extinction operates in a similar neurological manner as perceptual extinction, we hypothesized that during extinction recall the vmPFC would display greater activity for the CS+Ei compared to the CS+U.

## Materials and methods

### Participants

Twenty-eight undergraduate students (two males; mean age: 20.00, *SD *= 2.40) completed this study as part of a larger two-visit study. Visit 1 data were described in a previous publication (Jiang *et al*. 2021). Briefly, on Visit 1, participants underwent a separate differential fear conditioning paradigm, whereby participants were fear conditioned to different letter stimuli than those used in the present study. Additionally, mental imagery was not involved in any aspect of conditioning and there was no fear extinction or extinction recall manipulation. Both visits were independent. From Visit 1, participants for the current study were selected if they had a detectable SCR response to the US (i.e., mild shock) to ensure no data had to be excluded due to non-responders on Visit 2. Thus, the data presented here are from Visit 2 of the study and have not been published previously. For this study, one participant’s SCR data were removed from the Imagery Extinction phase, as well as another participant’s SCR data were removed from the Visual Extinction Recall phase (both due to technical difficulties). Analyses on all the other measures (i.e., self-reported fear, shock estimation, and fMRI) were based on the complete sample. This study was approved by the Institutional Review Board of the Louisiana State University (IRB 3847), and written informed consent was acquired from all participants prior to the beginning the experiment. All the data for the present study are made available at doi: 10.18112/openneuro.ds004407.v1.0.0.

### Materials

**Figure 1:**
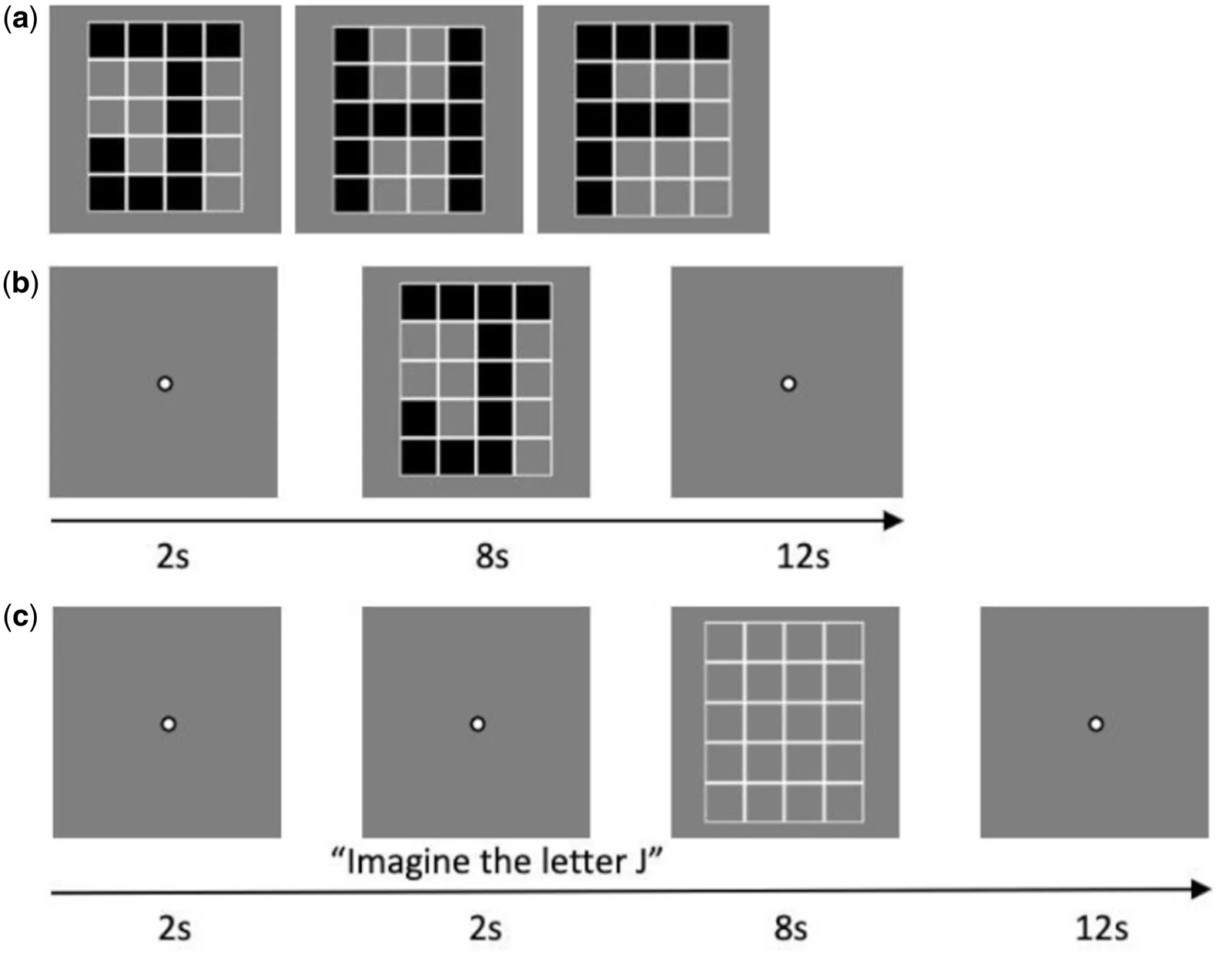
Paradigm (a), letter stimuli used; (b), an example of a visual trial as conducted in the Acquisition and Visual Extinction Recall phases. A trial began with a 2 s fixation, 8 s of viewing the letter stimulus on the grid, then ended with a 12 s inter-trial interval; (c) an example of an imagery trial as conducted in the Imagery Extinction Learning phase. A trial began with a 2 s fixation, then 2 s of audio instructions such as “Imagine the letter J,” 8 s of imagining the letter stimulus on the grid, then ended with a 12 s inter-trial interval.

A set of three letters (i.e., “J,” “H,” and “F,” see Fig. 1a) were used as the CSs. The US were 100-ms mild electric stimulations which were delivered to the distal phalanx of each participant’s ring finger and pinky on his/her non-dominant hand through attached electrodes. The mild electric stimulation used was customized to each participant, and a shock level was selected when it was reported to be “uncomfortable but not painful” and was administered through the STMISOC and STM100C modules of BIOPAC Systems.

### Procedure

**Figure 2:**
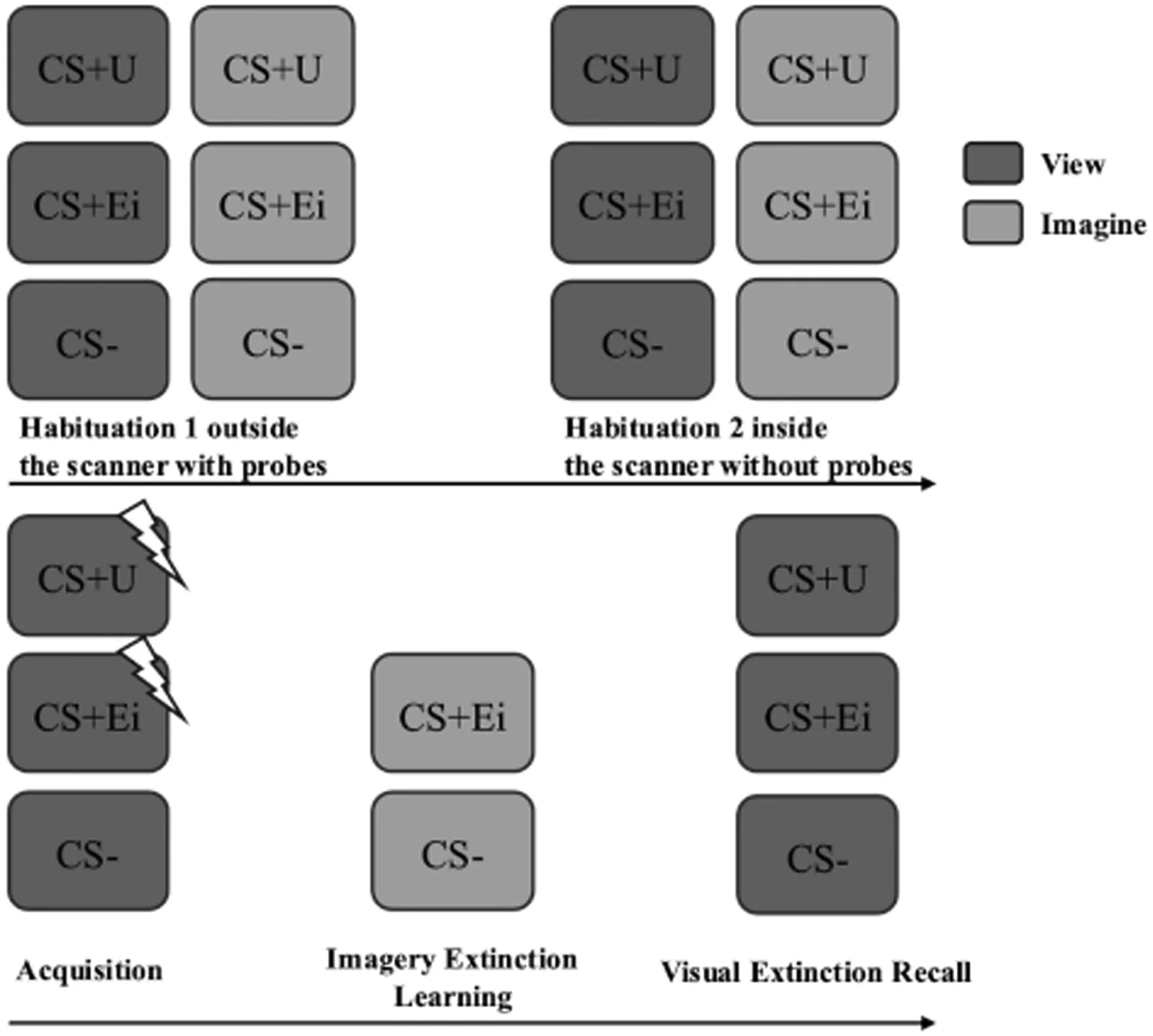
Study protocol. Illustration of each phase and the corresponding visual and imagery trials; CS+U = unextinguished CS+; CS+Ei = extinguished CS+ via mental imagery; CS− = CS that was never paired with shock. Dark gray boxes indicate visual trials; light gray boxes indicate imagery trials. The shock symbol indicates when stimuli were paired with shock.

The experimental paradigm and study protocol are illustrated in Figs 1 and 2, respectively. The study was composed of two habituations, one acquisition, and two extinctions in order. Before the habituations, participants completed demographic questions, including the Vividness of Visual Imagery questionnaire. In habituations, participants were instructed to view and imagine the CSs, i.e., “J,” “H,” and “F.” After completion of the first habituation (Habituation 1), which occurred outside the scanner, participants proceeded with the remaining procedures in the scanner starting with the second habituation (Habituation 2). In acquisition, visual images of the CSs were presented to the participants. Both CS+ (i.e., “J” and “H”) co-terminated with the US 50% of the time while the CS− (i.e., “F”) was never paired with the US. Following acquisition, all participants continued to the Imagery Extinction Learning phase in which they were instructed to imagine one of the two CS+ (i.e., CS+Ei; “J” or “H,” counterbalanced across participants) and the CS−. After, in the Visual Extinction Recall phase, all CSs were visually presented again.

A separate functional localizer task was added to the end of the study. Twenty fear and 20 neutral images were selected from the International Affective Picture System (Lang *et al*. 2008) and the Nencki Affective Picture System (Marchewka *et al*. 2014). All images were transformed into black and white and their low-level visual properties (i.e., luminance and contrast) were controlled using the SHINE Matlab toolbox (Willenbockel *et al*. 2010). The images were visually presented to each participant using a block design. Specifically, the functional localizer task had five fear blocks and five neutral blocks. Fear blocks contained four different negative images, and neutral blocks contained four different neutral images. The presentation was randomized both in terms of the block order and images within each block. Each block was presented for 10 s (2.5 s for each image) with intervals of 10 s between blocks. In total, the task took under 5 min to complete.

This localizer was added to identify emotional network activity in our sample. This allowed for specific regions-of-interest (ROIs) to be extracted from an independent dataset without inflating the false positive rate (Saxe *et al*. 2006). Additionally, we used negative affective images as our functional localizer to increase the generalizability of our findings (Sabatinelli *et al*. 2023). This approach identified ROIs more generally relevant to negative emotional reactivity, rather being overly specific to anticipation of electric shock.

### Probe

Probes were added to Habituation 1 outside the scanner. These probes were implemented to facilitate and improve the participants’ practice and help provide implicit feedback that they were imagining the letters correctly. No data were collected from these probes. A single square in the grid filled in in red appeared at a location randomly selected from a set of positions on the 4 x 5 grid was used as the probe. The participants were instructed to respond as quickly as possible by pressing 1 if the probe was on the letter presented/imagined or 2 if the probe was off the letter. A response window of 2 s was applied. Within each run, the probe had a 50% chance to be on the target letter. Probes were added to each trial at either 4, 5, or 6 s after stimulus onset.

### Physiological responses recording

Electrodermal activity was recorded with the Biopac MP-150 system and AcqKnowledge software (BIOPAC systems, Goleta, CA, USA) and was sampled at 2000 Hz. Analyses were carried out in MATLAB R2018a (Version 9.4) on SCR signals. A first-order Butterworth bandpass filter was applied with cut-off frequencies of .01 and 5 Hz (Bach *et al*. 2010). Time series were then down-sampled to 100 Hz. Trial-wise baseline detrending was conducted by subtracting the baseline (mean electrodermal activity recorded one second prior to CS onset) from the rest of the trial segment. Next, SCRs were calculated through a trough-to-peak analysis strategy. Specifically, a minimum SCR value was identified in the first second after CS onset. The maximum SCR value was identified between the 1–8 s time window after stimulus onset (Milad *et al*. 2007, Schiller *et al*. 2013, Grégoire and Greening 2019). An SCR that did not cross a 0.02 μs threshold was set to zero (Boucsein *et al*. 2012). To increase normality, the difference scores were then square root transformed (Burleigh *et al*. 2022). Shock trials were excluded as these SCRs may be more reflective of the unconditioned responses to the US instead of the conditioned ones to the CS (Greening *et al*. 2016), though these US trials were included as nuisance regressors in first level fMRI analyses (see below).

### Subjective ratings

Participants were surveyed on their fear of shock and the estimated percentage of shock paired with each CS after acquisition, extinction learning, and extinction recall. Participants reported their fear of each CS on a 7-point Likert scale (1 = “Not At All” to 7 = “Very Much So”). A 10-point Likert scale (0% to 100% with intervals of 10%) was used as estimations of shock contingency of each CS. Participants also provided self-reported evaluations of their vividness of the mental images on a 7-point scale ranging (1 = “Non-Existent” to 7 = “Very Strong”) and their effort when forming mental images on a 7-point scale ranging (1 = “Not At All” to 7 = “Very Hard”). Participants also reported their fear levels of each image presented after the functional localizer task. A 5-point Likert scale was used here (1 = “Not At All” to 5 = “Very Much So”).

### MRI data collection and analysis

#### MRI acquisition

Participants were scanned during the second visit of a two-visit procedure from the beginning of Habituation 2 to the end of the functional localizer. Imaging data were collected on a GE MR750w 3.0 T system with a 32-channel MR Instruments head coil at Pennington Biomedical Research Center, Baton Rouge, Louisiana. T1-weighted structural images were acquired using a three-dimensional fast spoiled gradient-echo sequence (time to repition = 8.7 ms, time to echo = 3.8 ms, flip angle = 8°, 256 × 256 matrix, phase encoding direction anterior to posterior, field of view = 25.6 cm). One hundred eighty sagittal slices covering the entire brain were acquired in sequential order producing a voxel resolution of 1 mm isotropic. T2*-weighted functional scans were acquired using gradient-echo echo-planar imaging (echo-planar imaging; TR = 2000 ms, TE = 25 ms, flip angle = 90°, 64 x 64 matrix, phase encoding direction anterior to posterior, FOV = 22.4 cm). Thirty-six axial slices covering the whole-brain were acquired with a voxel resolution of 3.5 mm isotropic with no gap. Slices were acquired in interleaved ascending order. Each functional scan began with three dummy volumes to account for equilibrium effects, and these dummy volumes were discarded from the analyses during preprocessing. The number of volumes varied for each portion of the experiment. Specifically, each habituation run had 130 volumes, each acquisition run had 124 volumes, extinction learning run had 136 volumes, each extinction recall run had 124 volumes, and the functional localizer run had 108 volumes.

#### fMRI preprocessing and whole-brain univariate analyses

fMRI data were analyzed using FEAT (FMRI Expert Analysis Tool) Version 6.0, part of FSL (FMRIB’s Software Library, www.fmrib.ox.ac.uk/fsl). Registration to participant structural and standard space images was carried out using FSL’s Linear Registration Tool (FLIRT) (Jenkinson and Smith 2001, Jenkinson *et al*. 2002). Pre-statistics processing applied motion correction using MCFLIRT (Jenkinson *et al*. 2002), slice-timing correction using Fourier-space time-series phase-shifting, non-brain removal using FSL’s Brain Extraction Tool (BET) (Smith 2002), spatial smoothing using a Gaussian kernel of FWHM 6 mm, grand-mean intensity normalization by a single multiplicative factor, and high-pass temporal filtering (Gaussian-weighted least-squares straight line fitting, with sigma = 50.0 s). The time-series modeling was carried out using FSL’s Improved Linear Model (FILM) with local autocorrelation correction (Woolrich *et al*. 2001).

At the single-subject level each run was modelled separately. A double-gamma hemodynamic response function convolution was used on each of the conditions of interest/explanatory variables (i.e., CS+Ei, CS+U, CS-, CS+Ei/US, CS+U/US) inputted in Custom (three-column format) basic shape, which is consistent with previous research and attempts to model both the activation and undershoot of the hemodynamic response (Greening *et al*. 2022). Temporal derivatives of each condition of interest were added. To reduce the impact of motion related artifacts, consistent with previous research (Burleigh and Greening 2023), we employed several nuisance regressors, including six original motion parameters, and framewise displacement (FD) = 0.9 mm motion censoring (Siegel *et al*. 2014) using the fsl_motion_outliers function. Relevant only to the acquisition phase, the CS+/US trials were also separately modeled to ensure the delivery of the US did not confound the estimation of the condition response. A second-level analysis was performed to average over contrast estimates from the first-level analysis for each experimental phase (e.g. acquisition) for each participant. As noted previously, US (i.e., CS+Ei/US, CS+U/US) trials were not used in higher-level analyses. These analyses were carried out using a fixed-effects model in FLAME (FMRIB’s Local Analysis of Mixed Effects), with the random effects variance forced to zero (Beckmann *et al*. 2003, Woolrich *et al*. 2004, Woolrich 2008). Group-level analyses were carried out using FLAME (FMRIB’s Local Analysis of Mixed Effects) stage 1 and 2 (Beckmann *et al*. 2003, Woolrich *et al*. 2004, Woolrich 2008). The resulting z (Gaussianized T/F) statistic images were corrected for multiple comparisons using FSL’s cluster thresholding algorithm that applies Gaussian random field theory to estimate the probability of observing clusters of a given size. As is now the default in FSL, we applied a cluster-forming threshold of z > 3.1 (one-tailed) and a (corrected) cluster size probability of *P *< 0.05 (Worsley 2012).

#### fMRI regions of interest

Similar to Sabatinelli *et al*. 2023, a brief functional localizer task was conducted to identify cortical and subcortical regions that are reliably found in emotional processing. This task allowed the ROI selection to be independent of the data being analyzed. Remaining consistent with the univariate analysis, the preprocessing and level 1 parameters stayed the same as described above for the main analyses. However, specific to the functional localizer, the group-level analysis used a fixed-effects model, thresholded at *z* > 3.1. The clusters that survived correction but were too large such that they encompassed more than one brain regions were first eroded to allow for their isolation, then were dilated to the same extent they were eroded. This approach allows for group-defined functional ROI creation without erroneously combining distinct regions into one ROI. When contrasting negative to neutral images, masks of the bilateral dACC, left AIC (L-AIC), right AIC (R-AIC), and the left and right amygdala were created. To create a mask of the vmPFC, we used the neutral greater than negative images contrast.

#### Psychophysiological interaction

We next conducted a psychophysiological interaction (PPI) analysis to assess functional connectivity differences in task-specific correlations between a seed region, the vmPFC, and other areas (O’Reilly *et al*. 2012) on the data from the Visual Extinction Recall phase. Specifically, we were interested in functional connectivity differences during CS+Ei versus CS+U between the vmPFC and the amygdala (Milad *et al*. 2007, Schiller *et al*. 2013). The seed region was produced from the vmPFC ROI derived from the functional localizer, but warped to each individual subject’s space.

At the single-participant (first-level) we ran a general linear model (GLM) that included: our psychological regressor contrasting our two conditions of interest, CS+Ei and CS+U; our physiological regressor, which was the time-series of the vmPFC extracted from the preprocessed and filtered data supplied to our initial first-level analyses; and the critical PPI regressor, which was modelled as the interaction between the first two regressors such that the physiological regressor zero centered on the mean (i.e., the “mean” option) and the psychological regressor zero centered on the halfway point between the highest and lowest point of the regressor (i.e., the “center” option). No temporal derivative or temporal filtering was applied to the PPI regressor. Our PPI model also included nuisance regressors which included one regressor reflecting the shared variance of the two PPI conditions of interest, i.e., CS+Ei + CS+U, all other condition regressors from the standard univariate model, six motion correction parameters, and motion censoring regressors (the same as described in the main level 1 analysis).

Two group-level analyses were carried out on the PPI parameter estimate using the same approach as deployed in the main group analysis described above. Namely, we used FLAME stage 1 and 2, and we applied a cluster-forming threshold of *z* > 3.1 (one-tailed) and a (corrected) cluster size probability of *P *< 0.05). The first PPI group-analysis was carried out using small volume correction by considering only voxels within the bilateral right and left amygdala ROIs from the functional localizer, which was the region of primary interest for this analysis (Agren *et al*. 2012, Schiller *et al*. 2013). This approach is common when interrogating the amygdala in studies of conditioning (Lonsdorf *et al*. 2014, Lally *et al*. 2017). Specifically, the cluster-forming threshold of *z* > 3.1 (one-tailed) and a (corrected) cluster size probability of *P *< 0.05 was restricted to only those voxels in the bilateral amygdala mask. The second group analysis was a whole-brain PPI analysis, which was conducted for exploratory purposes, consistent with the univariate whole-brain analysis.

#### Representational similarity analysis

For each participant, every trial was modelled separately with the same preprocessing steps as above. Further, the same ROI masks were used as above, with the L-AIC and R-AIC being combined to make the bilateral AIC mask. For each trial, a vector of the z-values from each voxel in each ROI mask was created. For each condition (CS+U, CS+Ei, CS−), the corresponding trial-wise *z*-values were averaged together for the Acquisition phase, the early and late portion of the Imagery Extinction Learning phase, and the early and late portion of the Visual Extinction Recall phase (Visser *et al*. 2011, Graner *et al*. 2020). Both shock trials, and the first and last CS− trials of a run were excluded to ensure an equal number of trials in each condition (Greening *et al*. 2022). This resulted in 13 vectors for each ROI mask. Next, a dissimilarity measure for each pairwise vector was created by correlating the vectors with one other and subtracting 1 from their Pearson *r* (1 − r; Kriegeskorte *et al*. 2008), per participant. These dissimilarity measures were then compared across phases, such that a 2 (Type: CS+Ei vs. CS−) x 2 (Time: Early vs. Late) ANOVA would assess the similarity of the Acquisition phase to the early and late portions of extinction (Graner *et al*. 2020). In so doing, one would expect high similarity for the CS+ at the beginning of the extinction phase to the Acquisition phase in fear network regions including the AIC and dACC (Hennings *et al*. 2022). However, as extinction occurs, we expected that the pattern of activity during the late portion of the extinction phase would become dissimilar to the Acquisition phase, reflecting new information relating to fear (Graner *et al*. 2020).

## Results

### Physiological and self-report data

#### Acquisition phase

**Figure 3:**
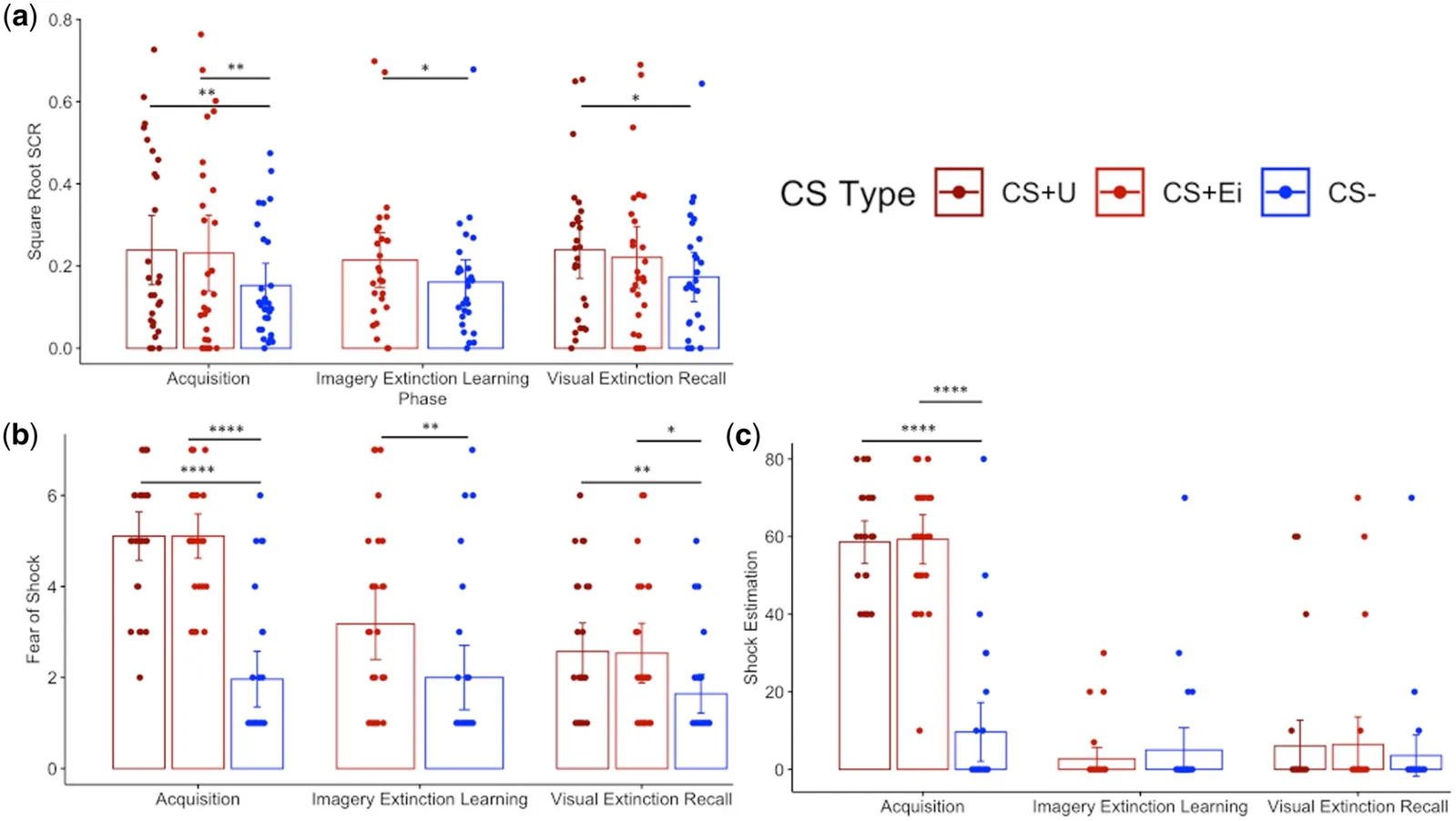
(a) Square root SCR (Y axis) by phase (X axis); (b) self-reported fear of shock ratings obtained after each phase; (c) shock estimation (Y axis: estimate percent of trials containing shock) obtained after each phase. CS+U = the unextinguished CS+ (furthest left bar; dark red; presented during the acquisition and visual extinction recall); CS+Ei = the CS+ that was extinguished via imagery during the Imagery Extinction Learning phase (middle bar; light red; presented during acquisition, imagined during imagery extinction learning, and presented in visual extinction recall); CS− = stimulus never paired with the US (furthest right bar; blue; presented during acquisition, imagined during imagery extinction learning, and presented in visual extinction recall). Error bars show 95% confidence intervals. Each dot represents one subject. *P < .05. **P < .01. ****P < .0001.

Acquisition was examined using repeated-measures analyses of variance (ANOVAs) with CS type (i.e., the unextinguished CS+, also known as, CS+U; the CS+ that was extinguished via mental imagery, also known as, CS+Ei; and, the CS that was never paired with shock, also known as, CS−) as the within-subject factor on SCR, self-reported fear, and shock estimation separately (Fig. 3). Significant findings were followed by post hoc paired *t-*tests without corrections (Saville 1990). These three ANOVAs returned consistent results with significant main effects of CS type on SCR, *F*(2, 54) = 8.135, p < .001, *η_p_*^2^ = .23, self-reported fear, *F*(2, 54) = 70.16, *p <* .001, *η_p_*^2^ = .72, and shock estimations, *F*(2, 54) = 77.58, *p* < .001, *η_p_*^2^ = .74. Post-hoc *t-*tests revealed differential conditioning for both the CS+U and CS+Ei on all three measures. Specifically, participants had larger SCR for the CS+U, *t*(27) = 3.38, *p* = .002, *d *= 0.64, and CS+Ei, *t*(27) = 2.84, *p* = .008, *d *= 0.54, compared to the CS−. They also had greater self-reported fear for the CS+U, *t*(27) = 9.46, *p* < .001, *d *= 1.79, and the CS+Ei, *t*(27) = 9.04, *p* < .001, *d *= 1.71 than the CS−. Higher likelihood of shock was reported for the CS+U, *t*(27) = 10.88, *p* < .001, *d *= 2.06, and the CS+Ei, *t*(27) = 9.22, *p* < .001, *d *= 1.74, than the CS−. The CS+U and CS+Ei did not differ for SCR, *t*(27) = 0.45, *p* = .654, *d *= 0.09; self-reported fear, *t*(27) < 0.01, *p *= 1.00, *d *= 0.0; or shock estimation, *t*(27) = 0.20, *p* = .847, *d *= 0.04. There was no interaction by Time and all analyses that considered Time as a factor can be found in the Supplementary Material.

#### Imagery Extinction Learning phase

To contrast the CS+Ei and CS−, paired *t-*tests were carried out on mean SCR, self-reported fear, and shock estimation on the Imagery Extinction Learning phase (Fig. 3). Results based on self-reported fear and SCR supported a transfer of differential fear from viewing to imagining the CS+Ei. Specifically, when imagined, the CS+Ei had greater SCR, *t*(26) = 2.35, *p* = .027, *d *= 0.45, and, self-reported fear, *t*(27) = 2.78, *p* = .010, *d *= 0.53, than the CS-. These elevations of subjective and physical fear responses were apparent despite participants’ accurate knowledge of shock estimations during the imagery extinction learning phase. Close to 0% of shock likelihoods were reported for both CS+Ei and CS− (Fig. 3c), and a paired *t-*test revealed no significant difference, *t*(27) = −1.06, *p* = .30, *d *= 0.20. There was no interaction by Time, early versus late (see the Supplementary Material, section 1.2).

#### Visual Extinction Recall phase

Similar to the Acquisition phase, extinction recall was examined using a one-way ANOVA with CS type (i.e., CS+U, CS+Ei, and CS−) as the within-subject factor on SCR, self-reported fear, and shock estimation separately (Fig. 3). While SCR, *F*(2, 52) = 4.30, *p* = .019, *η_p_*^2^ = .14, and self-reported fear, *F*(2, 54) = 6.49, *p* = .003, *η_p_*^2^ = .19, analyses both reported significant main effects of CS type, post-hoc analyses yielded somewhat different results. Based on self-reported fear data, differential fear was still apparent for both the CS+U and the CS+Ei during extinction recall. Specifically, participants reported higher levels of fear for the CS+U, *t*(27) = 3.42, *p* = .002, *d *= 0.65, and the CS+Ei, *t*(27) = 2.70, *p* = .012, *d *= 0.51, in contrast to the CS− with no difference between the CS+U and CS+Ei, *t*(27) = 0.13, *p* = .896, *d *= 0.03. Paired *t*-tests on mean SCR, however, provided evidence of greater fear only for the CS+U, *t*(26) = 2.55, *p* = .017, *d *= 0.49, and not for the CS+Ei, *t*(26) = 2.02, *p* = .054, *d *= 0.39, in contrast with the CS-. Again, the CS+U and CS+Ei did not differ, *t*(26) = 0.93, *p* = .359, *d *= 0.18. Shock estimations remained close to 0% for all stimuli, with no significant effect of CS type, *F*(2, 54) = 0.89, *p* = .418, *η_p_*^2^ = .03. There was no interaction by Time (see the Supplementary Material, section 1.3).

### Univariate region of interest analysis

#### Acquisition phase

**Figure 4:**
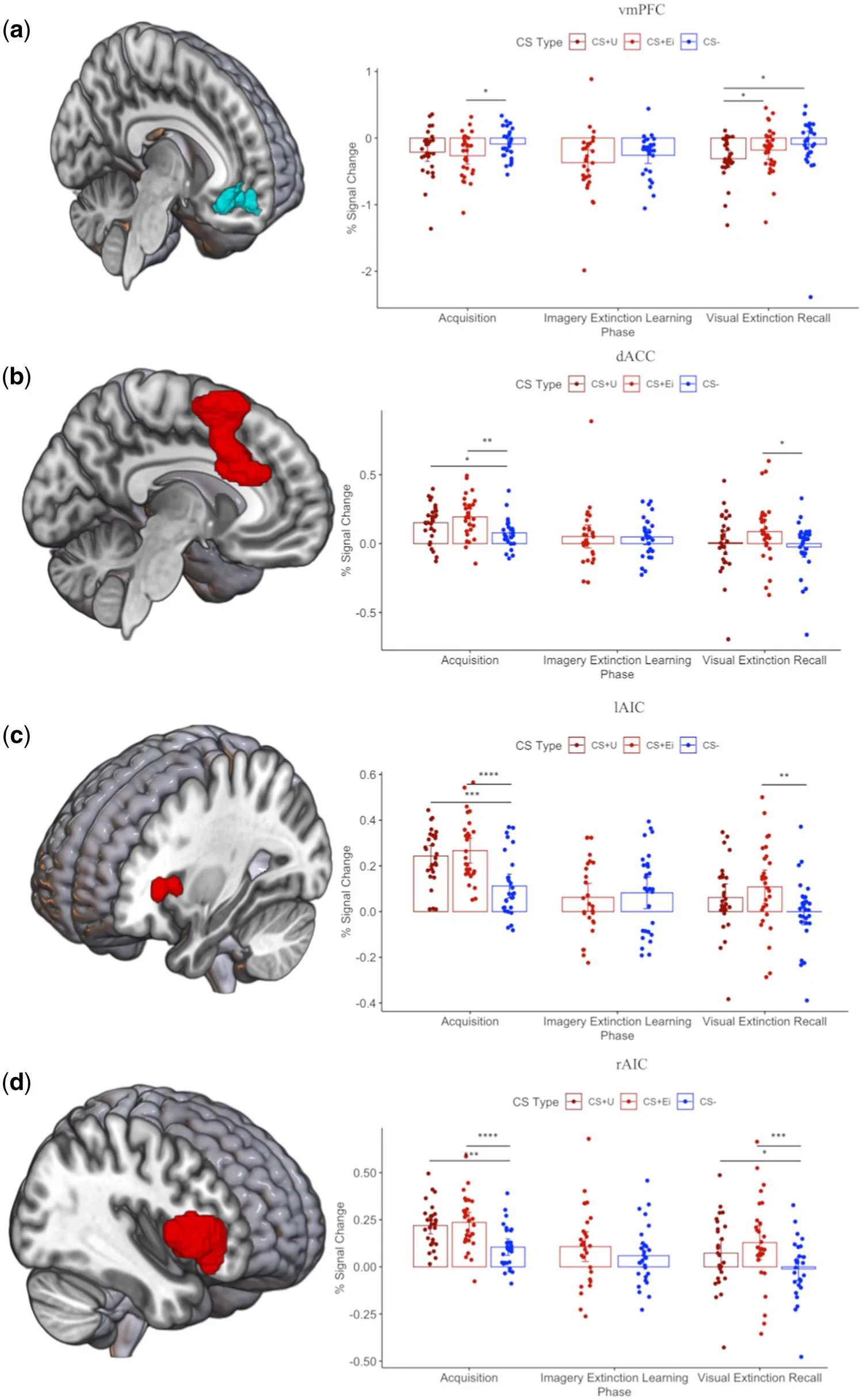
Results of ROI analyses of mean brain activations during each study phase in ROIs: vmPFC (a), bilateral dACC (b), L-AIC (c), and R-AIC (d). Error bars show 95% confidence intervals. Each dot represents one subject. *P < .05. **P < .01. ***P < .001. ****P < .0001.

ROI masks, derived from the functional localizer, were applied to univariate whole-brain data during acquisition. Consistent with the physiological and self-report analysis, the same one-way ANOVA with CS type (i.e., CS+U, CS+Ei, and CS−) being the within-subject factor was conducted on mean brain activations during acquisition within each ROI (Fig. 4). Significant main effects of CS type were found in four ROIs, including the vmPFC, *F*(2, 54) = 4.10, *p* = .022, *η_p_*^2^ = .13, bilateral dACC, *F*(2, 54) = 7.24, *p* = .002, *η_p_*^2^ = .21, L-AIC, *F*(2, 54) = 19.37, *p* < .001, *η_p_*^2^ = .42, and R-AIC, *F*(2, 54) = 15.59, *p* < .001, *η_p_*^2^ = .37. No significant main effect of CS type was found in either the R-amygdala, *F*(2, 54) = 0.68, *p* = .510, *η_p_*^2^ = .03, or the L-amygdala, *F*(2, 54) = 2.89, *p* = .064, *η_p_*^2^ = .10. Given the lack of differential learning in bilateral amygdala, the amygdala was not interrogated further during the other two phases. However, for each phase, interested readers can find a non-significant analysis of the amygdala by Time in the Supplementary Material, section 2.

Post-hoc *t*-tests provided support for both the CS+U and CS+Ei in the bilateral dACC, R-AIC, and L-AIC, but only for the CS+Ei in the vmPFC. Specifically, for the vmPFC, whereas significantly lower fear was observed for the CS+Ei, *t*(27) = 2.71, *p* = .012, *d *= 0.51, when compared to the CS−, the CS+U did not significantly differ from the CS−, *t*(27) = 2.00, *p* = .056, *d *= 0.38, or the CS+Ei, *t*(27) = 0.858, *p* = .398, *d *= 0.16. However, in the dACC, the CS+U, *t*(27) = 2.37, *p* = .024, *d *= 0.45, and the CS+Ei, *t*(27) = 3.46, *p* = .002, *d *= 0.65, both had greater activations than the CS-. No difference was found between the CS+U and CS+Ei, *t*(27) = 1.51, *p* = .144, *d *= 0.28. The same pattern was found in the L-AIC, CS+U vs. CS−: *t*(27) = 4.32, *p* < .001, *d *= 0.82; CS+Ei vs. CS−: *t*(27) = 5.36, *p* < .001, *d *= 1.01; CS+U vs. CS+Ei: *t*(27) = 1.19, *p* = .244, *d *= 0.26, as well as the R-AIC, CS+U vs. CS-: *t*(27) = 3.95, *p* < .001, *d *= 0.75; CS+Ei vs. CS-: *t*(27) = 4.74, *p* < .001, *d *= 0.90; CS+U vs. CS+Ei: *t*(27) = 0.88, *p* = .387, *d *= 0.17.

#### Imagery Extinction Learning phase

Paired *t-*tests were conducted on mean activations during the Imagery Extinction Learning phase within all four ROIs which compared the imagined CS+Ei and CS− (Fig. 4). No significant results were found in the four ROIs. In the vmPFC, CS+Ei showed no elevation compared to the CS−, *t*(27) = −1.41, *p* = .170, *d *= 0.27. The same was found in the dACC, *t*(27) = 0.05, *p* = .957, *d *= 0.01, the L-AIC, *t*(27) = -0.50, *p* = .624, *d *= 0.09, and the R-AIC, *t*(27) = 1.03, *p* = .314, *d *= 0.19, as the CS+Ei and CS− did not differ significantly from one another.

#### Visual Extinction Recall phase

The same ANOVAs applied to acquisition ROI analysis were repeated here (Fig. 4). There were significant main effects of CS type in the vmPFC, *F*(2, 54) = 4.26, *p* = .019, *η_p_*^2^ = .14, bilateral dACC, *F*(2, 54) = 3.51, *p* = .037, *η_p_*^2^ = .12, L-AIC, *F*(2, 54) = 5.65, *p* = .006, *η_p_*^2^ = .17, and R-AIC, *F*(2, 54) = 8.51, *p* < .001, *η_p_*^2^ = .24). Indicative of extinction recall, results of post-hoc *t*-tests revealed that in the dACC the CS+Ei, *t*(27) = 2.58, *p* = .015, *d *= 0.49, but not the CS+U, *t*(27) = 0.75, *p* = .457, *d *= 0.14, had a greater activation than the CS-. No difference was found between the CS+U and CS+Ei, *t*(27) = -1.76, *p* = .091, *d *= 0.33. A similar pattern was found in the L-AIC as the CS+Ei, *t*(27) = 3.27, *p* = .003, *d *= 0.62, but not the CS+U, *t*(27) = 1.97, *p* = .060, *d *= 0.37, had a greater activation than the CS−. Again, there was no difference between the CS+U and CS+Ei, *t*(27) = −1.44, *p* = .162, *d *= 0.27. Differing slightly, for the R-AIC, both the CS+U, *t*(27) = 2.54, *p* = .017, *d *= 0.48, and the CS+Ei, *t*(27) = 4.36, *p* < .001, *d *= 0.82, had greater activations than the CS−. No difference was found between the CS+U and CS+Ei, *t*(27) = −1.51, *p* = .142, *d *= 0.29.

Importantly, unlike the other ROIs, the pattern of activation for the vmPFC showed the CS+U, *t*(27) = −2.59, *p* = .015, *d *= 0.49, but not the CS+Ei, *t*(27) = −1.13, *p* = .269, *d *= 0.21, as being significantly lower from the CS−. Moreover, the CS+U had significantly less activation than the CS+Ei, *t*(27) = −2.12, *p* = .044, *d *= 0.40.

#### ROI analysis across phases

**Figure 5:**
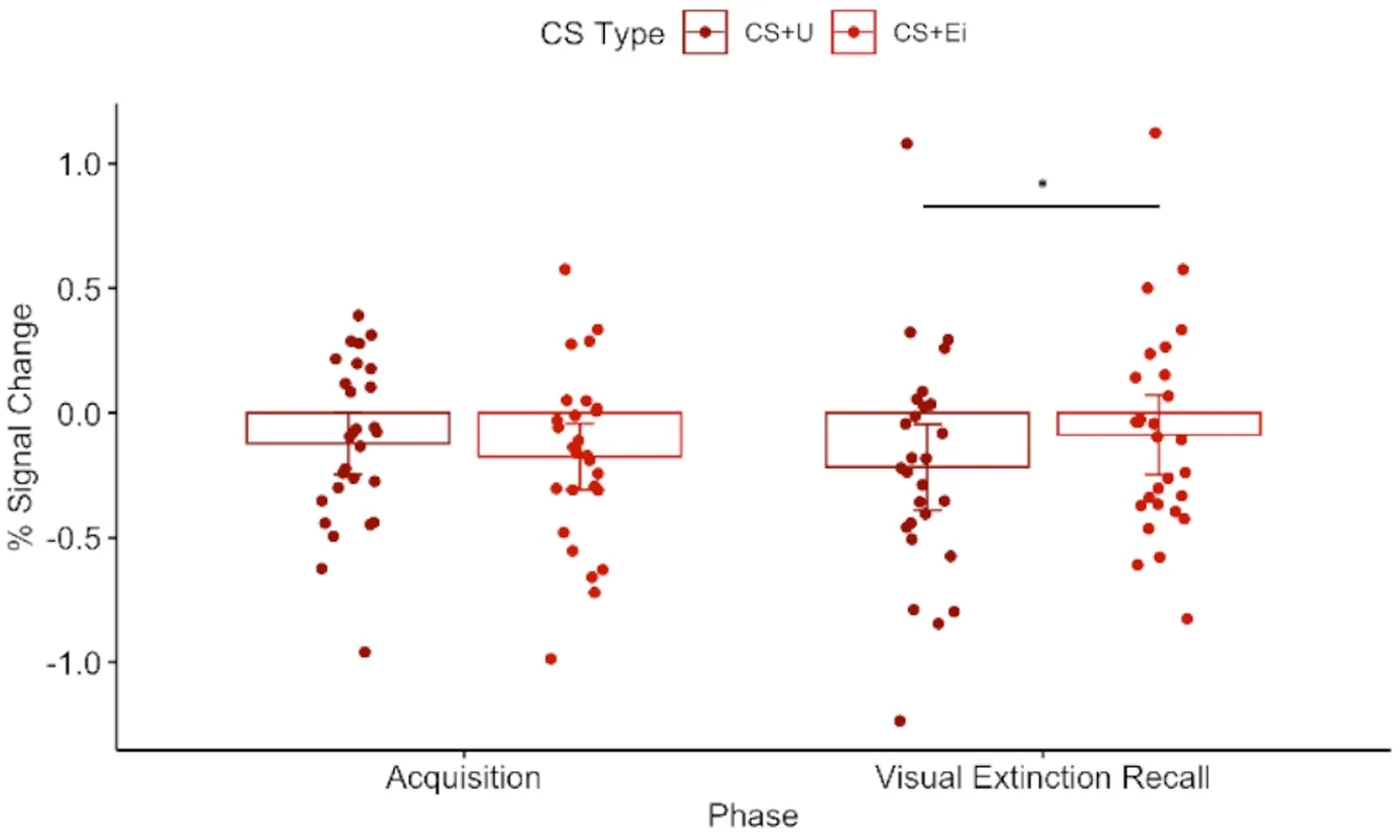
Results of the vmPFC analyses of mean brain activations during the Acquisition and Visual Extinction Recall phases. These data are the same as in Fig. 4 but with the CS− subtracted from both the CS+U and the CS+Ei, allowing for a direct comparison of the two CS+ across phases while controlling for the CS−. Error bars show 95% confidence intervals. Each dot represents one subject. *P < .05.

We next evaluated differential conditioning effects (i.e., CS+U—CS− and CS+Ei—CS−) across phases. Subtraction of the CS− was done from both the CS+U and CS+Ei of each phase, consistent with previous research investigating aspects of fear conditioning and extinction across phases (Phelps *et al*. 2004, Schiller *et al*. 2013). A 2 (CS type: CS+U vs. CS+Ei) x 2 (Phase: acquisition vs. visual extinction recall) ANOVA was conducted on each of the ROIs, while controlling for the CS− by subtracting its value from both the CS+U and CS+Ei (Fig. 5). In the vmPFC, there was a significant interaction between the CS+U and CS+Ei from the Acquisition to Visual Extinction Recall phase, *F*(1, 27) = 5.20, *p* = .031, *η_p_*^2^ = .16, no main effect of Type, *F*(1,27) = 0.65, *p* = .427, *η_p_*^2^ = .023, or Phase, *F*(1,27) = 0.002, *p* = .966, *η_p_*^2^ < .001, was found. For the dACC, no interaction was observed, *F*(1, 27) = 0.62, *p* = .439, *η_p_*^2^ = .02, as well as no main effect of Phase, *F*(1, 27) = 0.31, *p* = .583, *η_p_*^2^ = .01, however, there was a main effect of Type, *F*(1, 27) = 4.53, *p* = .043, *η_p_*^2^ = .14. Similar effects were found in the L-AIC and R-AIC. Specifically, no interactions were found in the L-AIC, *F*(1, 27) = 0.30, *p* = .586, *η_p_*^2^ = .01, or the R-AIC, *F*(1, 27) = 0.93, *p* = .345, *η_p_*^2^ = .03), nor were there main effects of CS type in the L-AIC, *F*(1, 27) = 4.14, *p* = .052, *η_p_*^2^ = .13, or R-AIC, *F*(1, 27) = 2.97, *p* = .096, *η_p_*^2^ = .10, or phase in the L-AIC, *F*(1, 27) = 2.03, *p* = .166, *η_p_*^2^ = .07, or R-AIC, *F*(1, 27) = 0.135, *p* = .717, *η_p_*^2^ = .01.

### Univariate whole-brain data

#### Acquisition phase

**Figure 6:**
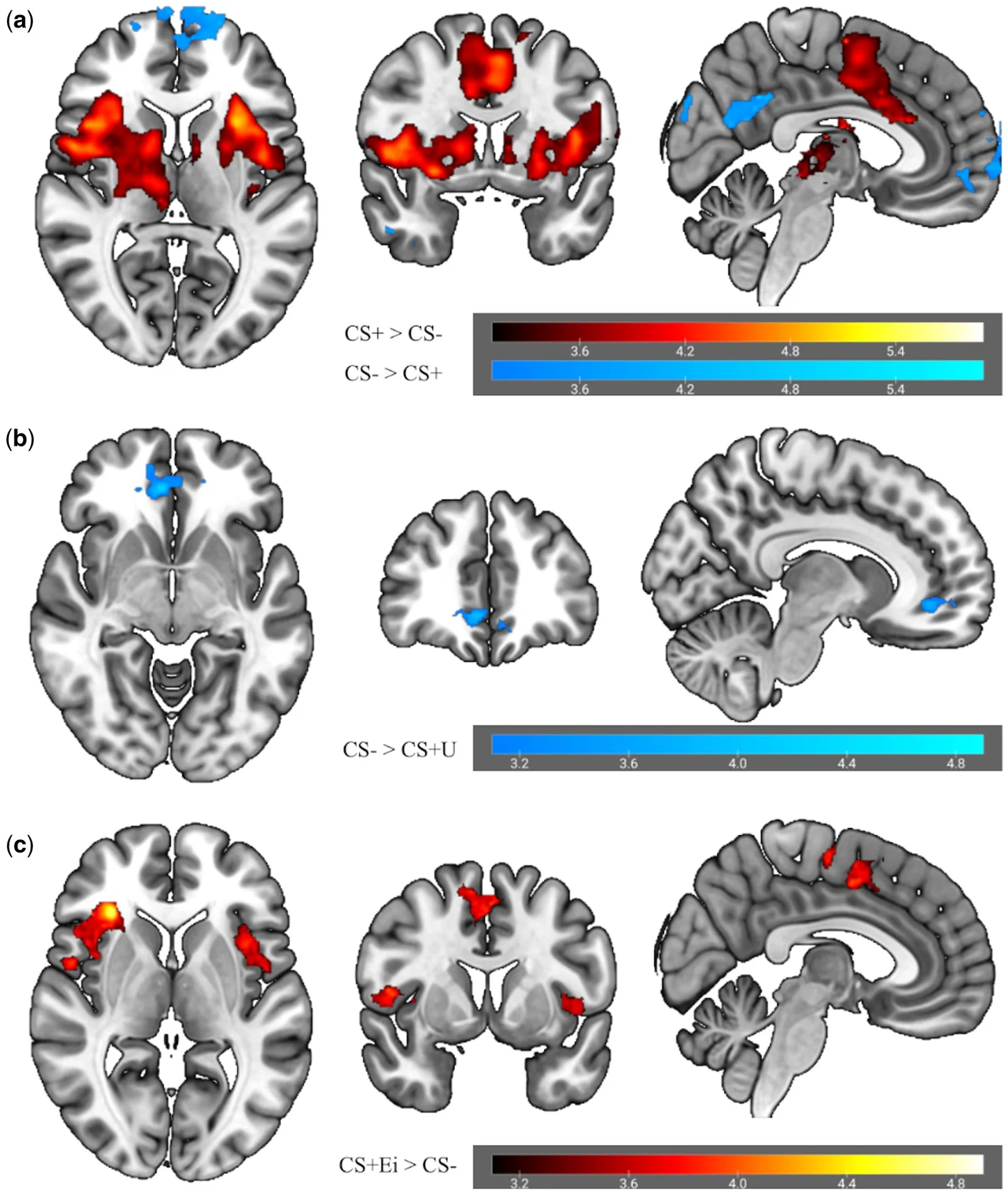
(a) Univariate whole-brain analysis results during acquisition comparing both CS+ to the CS−(red) and the CS− to both CS+ (blue); (b) Univariate whole-brain analysis results during visual extinction recall comparing the CS− to the CS+U (blue); (c) Univariate whole-brain analysis results during visual extinction recall comparing the CS+Ei to the CS− (red).

A network of regions associated with differential fear conditioning (Fullana *et al*. 2016) was found to exhibit significant functional activation during acquisition (Table 1). Specifically, whereas significantly greater signals were found in parts of bilateral AIC and bilateral dACC when viewing both the CS+U and CS+Ei compared to the CS−, significantly greater signals were found in the vmPFC when contrasting the CS− to both the CS+U and CS+Ei (Fig. 6a).

**Table 1:** Expression of fear conditioning during the acquisition phase.

						MNI		
Cluster #	Cluster k	Brain region	H	Z	*#*	*x*	*y*	*z*
**CS+ > CS−**								
1	12 546	Putamen	R	5.02	598	28	6	−4
		Thalamus	R	5.33	534	4	−16	14
		Putamen	L	4.92	469	−28	−2	−2
		Cingulate gyrus anterior division	R	4.73	314	2	4	34
		Insular cortex	L	4.98	309	−38	−12	−4
		Central opercular cortex	L	4.76	278	−44	2	10
		Supplementary motor cortex	R	4.90	237	4	−6	62
		Cingulate gyrus anterior division	L	6.11	232	0	6	36
		Supplementary motor cortex	L	4.96	220	−6	0	54
		Insular cortex	R	4.71	197	34	18	4
		Central opercular cortex	R	5.01	174	54	−2	10
		Supramarginal gyrus anterior division	L	3.89	170	−62	−30	26
		Precentral gyrus	L	3.95	167	−58	6	28
		Caudate	R	4.36	147	12	14	8
		Pallidum	R	4.13	142	20	−10	2
		Parietal operculum cortex	L	4.76	126	−52	−32	22
		Frontal operculum cortex	L	4.90	95	−32	18	10
		Thalamus	L	4.58	95	−6	−2	6
		Pallidum	L	4.21	93	−24	−4	−4
		Frontal operculum cortex	R	4.80	74	40	16	8
		Brain stem	R	4.60	60	4	−30	−6
		Precentral gyrus	R	4.22	57	58	8	8
		Postcentral gyrus	L	4.26	53	−64	−22	24
		Paracingulate gyrus	R	3.82	52	8	10	46
		Paracingulate gyrus	L	4.09	47	−10	18	40
		Brain stem	L	3.55	25	0	−28	−6
2	1290	Parietal operculum cortex	R	4.31	107	52	−22	20
		Supramarginal gyrus anterior division	R	6.33	70	66	−24	24
		Central opercular cortex	R	4.14	32	52	−14	12
**CS− > CS+**								
1	2019	Frontal pole	L	5.61	652	−8	62	26
		Frontal pole	R	5.41	295	4	66	12
		Paracingulate gyrus	L	4.46	33	−6	48	−6
		Paracingulate gyrus	R	4.09	33	12	50	0
		Frontal medial cortex	L	4.43	26	0	52	−12
2	1226	Lateral occipital cortex superior division	L	5.93	794	−46	−66	46
3	1129	Precuneous cortex	L	5.22	179	0	−68	26
		Precuneous cortex	R	5.29	161	6	−52	34
		Cingulate gyrus posterior division	L	5.10	69	−2	−50	30
		Cingulate gyrus posterior division	R	5.49	63	4	−50	34
4	882	Middle frontal gyrus	L	4.31	55	−32	22	52
		Superior frontal gyrus	L	3.76	33	−4	46	42
		Frontal pole	L	3.77	29	−16	42	50
5	551	Frontal pole	R	4.43	302	46	48	−2
		Frontal orbital cortex	R	4.05	70	22	32	−18
6	439	Temporal pole	R	5.07	228	48	8	−36
7	425	Frontal pole	R	5.43	51	20	42	44
		Superior frontal gyrus	R	5.63	33	22	28	56
8	346	Occipital pole	L	4.13	35	−2	−92	24
		Cuneal cortex	L	4.11	34	−2	−86	22
		Cuneal cortex	R	4.19	27	2	−84	32
9	283	Frontal pole	L	4.57	163	−44	50	−12
10	237	Temporal pole	L	4.59	177	−48	14	−36

Cluster # = the number of a cluster, ordered by size; Cluster k = the number of contiguous voxels in the cluster; Brain region = the region of local maxima included in the broader cluster. The region names are taken from the Harvard Oxford atlas in FSL; H = principal hemisphere of the cluster, right (R) or left (L); Z = maximum *z*-value from the cluster within the given brain region; *#* = number of voxels from the cluster inside the given brain region. Regions with less than 20 voxels in the cluster are not reported; MNI(x, y, z) = coordinates of the voxel with the maximum effect in the standardized space of the Montreal Neurological Institute (MNI), represented in units of 2 mm.

#### Imagery Extinction Learning phase

Univariate whole-brain analysis contrasting imagining the CS+Ei and CS− during the Imagery Extinction Learning phase yielded no significant findings.

#### Visual Extinction Recall phase

Notably, analysis during the Visual Extinction Recall phase found significant activation in the vmPFC when viewing the CS− compared to viewing the CS+U (Fig. 6b). Furthermore, consistent with the extinction recall findings from Fullana *et al*. (2018), greater activation was found in the bilateral AIC and bilateral dACC for the previously imagined CS+Ei compared to the CS− (Fig. 6c). No difference was found when contrasting the CS+U to the CS+Ei.

### PPI, amygdala ROI

The PPI analysis focused on the connectivity of the vmPFC and amygdala during the Visual Extinction Recall phase. This analysis compared the visual CS+Ei to the visual CS+U. After small volume correction to the bilateral amygdala, results suggested significantly greater connectivity between the vmPFC seed region and left amygdala [MNI: *x* −30, *y* −2, *z* −16; Max *z*-value = 3.34, cluster size = 18 voxels] and right amygdala [MNI: *x* 20, *y* 0, *z* −2; Max *z*-value = 3.50, cluster size = 15 voxels], for the CS+U as compared to the CS+Ei (Table 2).

**Table 2:** Results of the whole-brain PPI analysis with the vmPFC seed in the visual extinction recall phase.

Cluster #	Cluster k	Brain region	H	Z	#	X	Y	Z
**Visual extinction recall CS+Ei > CS+U**								
1	8392	Lateral occipital cortex inferior division	L	5.634883	200	−36	−84	4
		Lateral occipital cortex inferior division	R	4.591408	198	38	−84	2
		Lateral occipital cortex superior division	L	5.192571	64	−30	−86	16
		Lateral occipital cortex superior division	R	5.648237	73	34	−86	16
		Lingual gyrus	L	5.397128	54	−6	−88	−8
		Lingual gyrus	R	4.649560	31	6	−86	−8
		Occipital fusiform gyrus	L	5.638400	182	−20	−88	−14
		Occipital fusiform gyrus	R	5.927066	181	24	−82	−10
		Occipital pole	L	6.923065	893	−12	−100	4
		Occipital pole	R	6.889034	761	16	−98	10
**Visual extinction recall CS+U > CS+Ei**								
1	585	Frontal pole	L	3.719547	52	−14	42	50
		Middle frontal gyrus	L	3.470250	10	−30	28	50
		Superior frontal gyrus	L	3.337890	2	−20	32	42
2	1236	Angular gyrus	L	3.694555	24	−46	−60	24
2		Lateral occipital cortex inferior division	L	3.573795	23	−44	−70	12
2		Lateral occipital cortex superior division	L	4.612024	651	−42	−78	−30
3	1357	Angular gyrus	R	3.673349	13	56	−58	26
3		Lateral occipital cortex Inferior Division	R	3.776523	107	58	−66	6
3		Lateral occipital cortex superior division	R	4.677767	524	52	−74	24
4	1524	Accumbens	L	3.549144	4	−8	14	−8
		Cingulate gyrus anterior division	L	3.401613	6	−6	38	−4
		Cingulate gyrus anterior division	R	3.551594	10	4	42	−2
		Frontal medial cortex	L	3.920130	73	−8	44	−12
		Frontal medial cortex	R	3.982574	54	2	54	−12
		Frontal pole	L	3.875076	158	−6	60	−10
		Frontal pole	R	3.862085	70	2	56	−12
		Paracingulate gyrus	L	3.926700	126	−6	46	−8
		Paracingulate gyrus	R	3.684458	64	8	44	−6
		Subcallosal cortex	L	3.756995	68	0	18	−2
		Subcallosal cortex	R	3.682188	35	2	20	−4
5	2704	Cingulate gyrus posterior division	L	3.620748	53	−4	−54	24
		Cingulate gyrus posterior division	R	3.401222	20	10	−50	32
		Lingual gyrus	L	3.329233	5	−10	−50	−2
		Lingual gyrus	R	3.650225	64	14	−62	−8
		Postcentral gyrus	R	3.653676	9	4	−40	62
		Precentral gyrus	L	3.808301	57	−4	−24	66
		Precentral gyrus	R	3.642130	68	4	−26	68
		Precuneous cortex	L	3.990885	627	−4	−54	44
		Precuneous cortex	R	3.743488	486	10	−56	60

Cluster # = the number of a cluster, ordered by size; Cluster k = the number of contiguous voxels in the cluster; Brain region = the region of local maxima included in the broader cluster. The region names are taken from the Harvard Oxford atlas in FSL; H = principal hemisphere of the cluster, right (R) or left (L); Z = maximum *z*-value from the cluster within the given brain region; *#* = number of voxels from the cluster inside the given brain region. Regions with less than 20 voxels in the cluster are not reported; MNI(X, Y, Z) = coordinates of the voxel with the maximum effect in the standardized space of the Montreal Neurological Institute (MNI), represented in units of 2 mm.

### PPI, exploratory whole-brain

**Figure 7:**
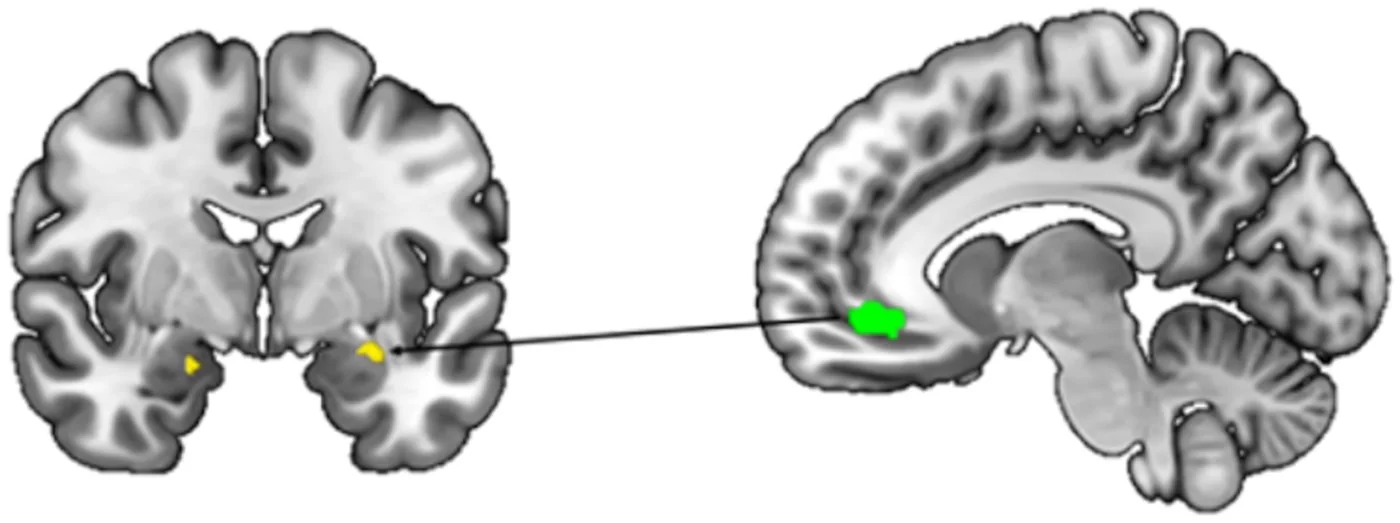
Functional connectivity results from the visual extinction recall phase revealed significantly greater connectivity between the vmPFC seed and bilateral amygdala while viewing CS+U compared to viewing the CS+Ei. These results are from the PPI analysis focused on the bilateral amygdala ROI with small volume correction.

Using the vmPFC as the seed region, a whole-brain analysis was conducted (Fig. 7). When comparing the CS+U to the CS+Ei, there was significantly increased strength of activity in a multitude of regions, as depicted in Table 2. Conversely, when comparing the CS+Ei to the CS+U, there was significantly increased strength of activity in occipital regions of the brain, as found in Table 2.

### Representational similarity analysis, exploratory ROI

#### Imagery Extinction Learning phase

**Figure 8:**
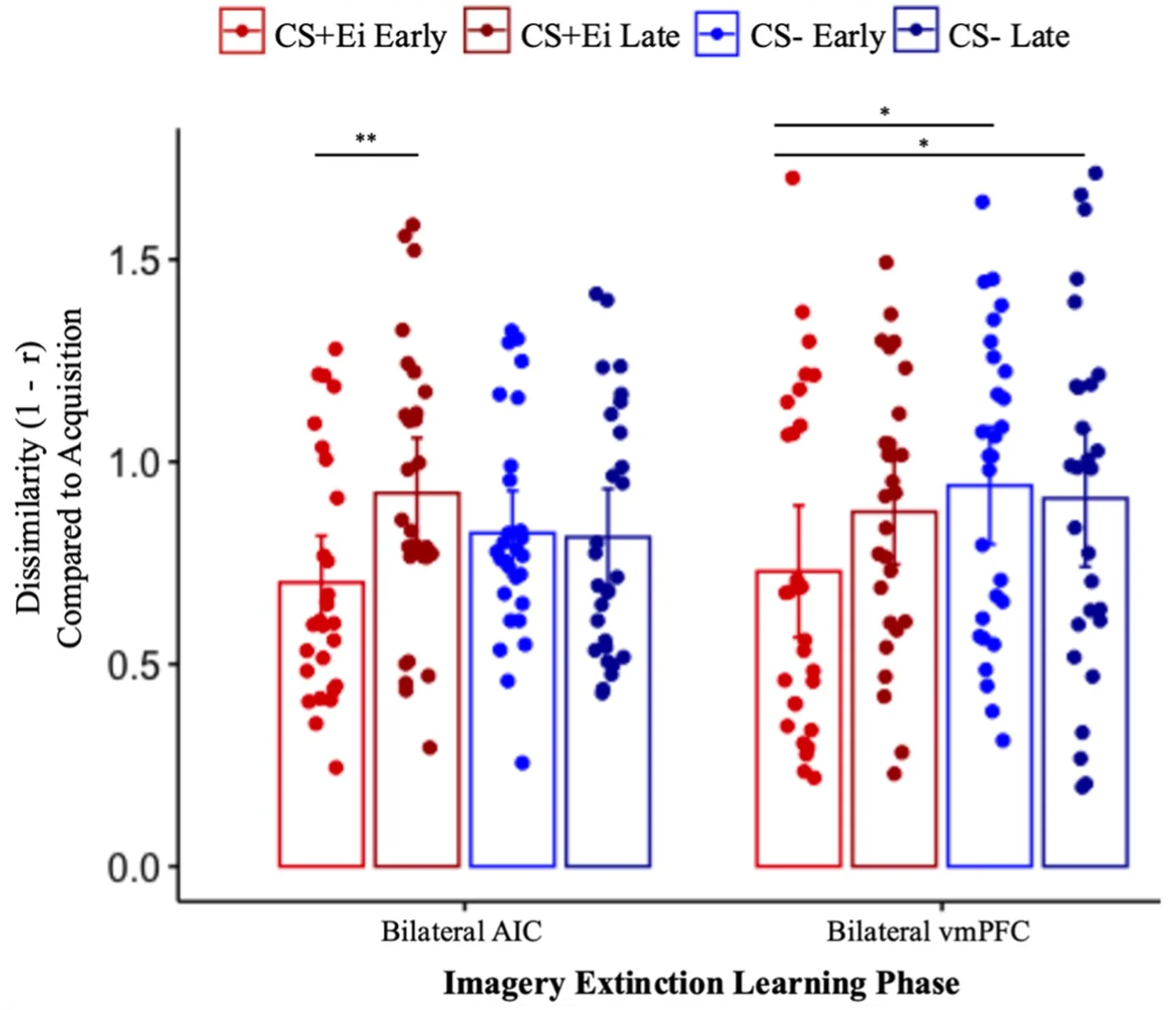
Representational similarity analysis for the Imagery Extinction Learning phase compared to the Acquisition phase for each condition. CS+Ei Early: first half of CS+Ei trials during the Imagery Extinction Learning phase; CS+Ei Late: second half of CS+Ei trials during the Imagery Extinction Learning phase; CS− Early: first half of CS− trials during the Imagery Extinction Learning phase; CS− Late: second half of CS− trials during the Imagery Extinction Learning phase. Error bars show 95% confidence intervals. Each dot represents one subject. The *y*-axis represents how dissimilar the pattern of activity was for each condition from the early and late portions of the Imagery Extinction Learning phase compared to the average pattern in the Acquisition phase of the respective condition. 0 represents perfect similarity, 1 represents a zero correlation, and 2 represents perfect dissimilarity. *P < .05. **P < .01.

To assess the representational similarity at the level of the voxel, we conducted an exploratory analysis of the bilateral AIC, vmPFC, and dACC (Fig. 8). In this analysis, the pattern of activity in each ROI during the Imagery Extinction Learning phase was compared to the pattern of activity during the Acquisition phase. This allowed for the quantification of the change in the informational content of each ROI across extinction learning with respect to the previous fear learning. A 2 (Type: CS+Ei vs. CS-) x 2 (Time: Early vs. Late) repeated measures ANOVA was conducted. An interaction effect was found in the bilateral AIC, *F*(1, 27) = 5.22, *p* = .030, *η_p_*^2^ = .16. There was no significant main effect of Type, *F*(1, 27) = 0.01, *p* = .917, *η_p_*^2^ < .01; or Time, *F*(1, 27) = 4.20, *p* = .050, *η_p_*^2^ = .14. Paired sample *t*-tests revealed that the differential in similarity between the early and late portion of the Imagery Extinction Learning phase drove this interaction, as the CS+Ei became significantly dissimilar from acquisition over the course of the phase, *t*(27) = −2.91, *p* = .007, *d *= 0.55; while the CS− did not change in similarity, *t*(27) = 0.15, *p* = .885, *d *= 0.03.

While not demonstrating an interaction effect, *F*(1, 27) = 1.57, *p* = .221, *η_p_*^2^ = .06, nor a main effect of either Type, *F*(1, 27) = 3.71, *p* = .065, *η_p_*^2^ = .12, or Time, *F*(1, 27) = 0.72, *p* = .404, *η_p_*^2^ = .03, the vmPFC did show significant differences among paired sample *t*-tests. Specifically, the CS+Ei was significantly dissimilar from the CS− during the early portion of the phase, *t*(27) = −2.25, *p* = .033, *d *= 0.43. This difference then dissipated when comparing the CS+Ei to the CS− during the late portion of the phase, *t*(27) = −0.34, *p* = .733, *d *= 0.06. There was no significant difference for the CS+Ei when comparing the early to late portion of the phase, *t*(27) = −1.63, *p* = .116, *d *= 0.31.

Similar to the vmPFC, the dACC did not show an interaction effect, *F*(1, 27) = 0.67, *p* = .420, *η_p_*^2^ = .02, nor a significant main effect of Type, *F*(1, 27) = 0.05, *p* = .832, *η_p_*^2^ < .01, or Time, *F*(1, 27) = 3.78, *p* = .062, *η_p_*^2^ = .12. Diverging from the vmPFC results, the dACC did not show any pairwise differences between the CS+Ei and the CS− during the early portion of the phase, *t*(27) = −0.79, *p* = .439, *d *= 0.15, or the late portion of the phase, *t*(27) = 0.38, *p* = .705, *d *= 0.07. Further, the dACC did not show a difference for the CS+Ei from the early to late portions of the phase, *t*(27) = −1.77, *p* = .089, *d *= 0.33.

#### Visual Extinction Recall phase

A 3 (Type: CS+U vs. CS+Ei vs. CS-) x 2 (Time: Early vs. Late) ANOVA was conducted on the Visual Extinction Recall phase for the same three ROI. For the AIC, there was no interaction effect, *F*(2, 54) = 0.45, *p* = .638, *η_p_*^2^ = .02, nor a main effect of Time, *F*(1, 27) = 3.60, *p* = .069, *η_p_*^2^ = .12; however, there was a main effect of Type, *F*(2, 54) = 4.10, *p* = .022, *η_p_*^2^ = .13. When interrogating this main effect of Type with paired sample *t*-tests, both the CS+U, *t*(27) = −2.25, *p* = .033, *d *= 0.42, and the CS+Ei, *t*(27) = -2.55, *p* = .017, *d *= 0.48, were significantly greater than the CS- during the early portion of the phase. Both of these differences from the CS+U, *t*(27) = −1.36, *p* = .186, *d *= 0.26, and CS+Ei, *t*(27) = −1.26, *p* = .218, *d *= 0.24, in comparison to the CS- then dissipated during the late portion of the phase. Similarly, for the vmPFC, there was no interaction, *F*(2, 54) = 0.04, *p* = .962, *η_p_*^2^ < .01, no main effect of Type, *F*(2, 54) = 0.09, *p* = .916, *η_p_*^2^ < .01, nor a main effect of Time, *F*(1, 27) = 3.57, *p* = .070, *η_p_*^2^ = .12. Further, there were no significant pairwise differences. Finally, for the dACC, there was no interaction, *F*(2, 54) = 0.05, *p* = .947, *η_p_*^2^ < .01, nor a main effect of Type, *F*(2, 54) = 2.00, *p* = .145, *η_p_*^2^ = .07; however, there was a significant main effect of Time, *F*(1, 27) = 6.62, *p* = .016, *η_p_*^2^ = .20. There were no significant pairwise differences.

## Discussion

The current study sought to assess the hypothesis that repeated mental imagery of a CS+ could replicate the results of perceptual extinction studies which compared the CS+E to CS+U during extinction recall. Supported by elevations in SCR and self-reported fear, we observed that both the unextinguished CS+ (CS+U) and the CS+ that was extinguished via mental imagery (CS+Ei) acquired differential fear. Furthermore, consistent with findings by Fullana *et al*. (2016), we found evidence of differential fear for both CS+ in the bilateral AIC and bilateral dACC. During the Imagery Extinction Learning phase, this conditioned fear then transferred to mental imagery as captured by greater SCR coupled with greater self-reported fear for the CS+Ei compared to the CS−. However, there was no difference observed between the CS+Ei and CS− in the vmPFC, which may be inconsistent with past perceptual research (Milad *et al*. 2007, Fullana *et al*. 2018). Nevertheless, upon re-exposure to viewing the CSs in the Visual Extinction Recall phase, SCR results indicated that only the CS+U, but not the CS+Ei, exhibited differential fear toward the CS−. However, the CS+Ei was marginally significant when compared to the CS−. Further confirmation was found for extinction recall when observing the vmPFC. Specifically, consistent with the recall of an extinction memory, the vmPFC demonstrated that the CS+Ei had significantly greater activation than the CS+U. This finding suggests that imagery exposure to the CS+Ei during extinction learning may have facilitated a new memory, whereby, similar to perceptual extinction, the CS+Ei may have become a safety signal, akin to the CS− (Fullana *et al*. 2018). Moreover, findings in the bilateral AIC and dACC both showed no difference between the CS+Ei and CS+U, with the CS+Ei being greater than the CS−. These results suggest that while imagery extinction learning may display weaker results compared to perceptual extinction learning, the following extinction recall has similar patterns of activation as found in perceptual extinction research (Milad *et al*. 2004, Phelps *et al*. 2004, Milad *et al*. 2007, Delgado *et al*. 2008, Fullana *et al*. 2018, Reddan *et al*. 2018).

Across measures, results from the Acquisition phase were largely consistent with previous fear conditioning literature (Phelps *et al*. 2004, Milad *et al*. 2007, Schiller and Delgado 2010). Both the physiological and subjective measures (i.e., SCR, self-report) demonstrated greater activation for both CS+ in comparison to the CS−, suggesting that fear had been successfully acquired (Milad *et al*. 2007). The fMRI data complemented these findings by showing increased activity toward the CS+ versus the CS− in the bilateral dACC and bilateral AIC, both of which have been implicated in fear acquisition (Fullana *et al*. 2016). We also found no significant activation of the amygdala, consistent with the previous literature (Fullana *et al*. 2016). The notable and expected difference to this pattern was found in the vmPFC, whereby both CS+ had lower, rather than higher, activity than the CS−. However, whereas the CS+Ei was significantly lower than the CS−, the CS+U was marginally so (*p* = .056). Overall, the general pattern of effect in the vmPFC is consistent with prior fear conditioning research (Milad *et al*. 2007), as during the process of fear conditioning, a suggested main function of the vmPFC is its ability to differentiate safety (CS−) from threat (CS+; Harrison *et al*. 2017, Fullana *et al*. 2018). Notably, while in the present study both CS+ were individually compared to the CS−, in the previous literature both the CS+ were combined and compared to the CS− using a single comparison (Milad *et al*. 2007). Nevertheless, during the subsequent visual extinction recall, the CS+U showed significantly lower activation than the CS− in the vmPFC. This finding, in combination with greater SCR and self-reported fear between the CS+U and CS− in extinction recall, suggests a persistence of differential fear between the CS+U and CS− from the acquisition phase.

In the Imagery Extinction Learning phase, elevations in SCR and self-reported fear to the CS+Ei remained higher than the CS−, suggesting that the visually acquired fear transferred to the imagery of the CS+Ei and CS−. These findings replicate the transfer of conditioned fear from perception to imagery and vice versa (Agren *et al*. 2017, Reddan *et al*. 2018, Jiang and Greening 2021, Greening *et al*. 2022, Burleigh and Greening 2023, Lyons *et al*. 2024). However, this difference between the CS+Ei and CS− did not fully dissipate by the end of extinction, as is typically found. This finding could suggest that imagined extinction may be weaker than its perceptual counterpart. Future research could examine whether more imagery extinction trials are necessary in comparison to visual extinction trials. Additionally, throughout this phase, none of the ROIs demonstrated a significant univariate difference between the CS+Ei and CS−. Past research has shown that particularly in the vmPFC, equivalent activation for the CS+ and CS− is not an uncommon finding (Fullana *et al*. 2018). Indeed, as the CS+ undergoes a change from threat to safety, it can be difficult to detect univariate differences. To alleviate this issue, multivariate analysis can be utilized. In the present study, when using an exploratory multivariate approach (Graner *et al*. 2020), subtle changes in the representational content were observed in the patterns of activity for the AIC and vmPFC throughout extinction learning. Exemplified in the bilateral AIC, the representational similarity from the Acquisition phase was greater for the CS+Ei at the beginning of the phase. When participants imagined the CS+Ei devoid the US, the CS+Ei lost its similarity to the Acquisition phase. In comparison, the representation of the CS− was unaffected throughout extinction. This interaction is consistent previous research (Graner *et al*. 2020) and suggests that extinction learning occurred. Importantly, in Graner *et al*. (2020), participants were visually presented the stimuli during extinction learning. To our knowledge, the current study is the first to find similar results when using imagery to extinguish a visually acquired fear. Additional evidence for extinction learning was found in the vmPFC, as the representational similarity was greater for the CS+Ei in comparison to the CS− during the early portion of the phase. This difference then dissipated during the late portion of the phase. However, as there was no interaction effect for the vmPFC, caution may be advised when interpreting these RSA results. Nevertheless, while supporting the use of imagery as an extinction strategy, these findings build on a growing literature that describes imagery as being an attenuated form of perception (Pearson 2019). As well, these results demonstrate the utility of multivariate analysis to discover nuanced changes in representation that univariate analyses may miss.

During the Visual Extinction Recall phase, support was found for the main research question as SCR findings demonstrated no significant difference between the CS+Ei and the CS− but did show a persistent difference between the CS+U and the CS−. However, no difference was found between the critical comparison of the CS+Ei and CS+U. Caution may be advised when interpreting the comparisons of either CS+ to the CS−, as there was only one CS− that was both visually presented and imagined, which may have confounded, for example, the CS+U vs CS− comparison. Future research may benefit from a factorial design that incorporates another CS− that is never imagined. Additionally, the non-significant SCR result of the CS+Ei versus CS− was *p* = .054, which could be considered marginally significant. Nevertheless, these findings suggest that imagined extinction, akin to perceptual extinction, facilitated a physiological dampening of the fear response (Reddan *et al*. 2018). While these results are encouraging, somewhat contradictory findings were observed for self-reported fear, as both the CS+Ei and CS+U remained higher in comparison to the CS−. A persistence of self-reported differential conditioning following both imagery and visual extinction has been reported previously (Lau *et al*. 2008, Shechner *et al*. 2015, Jiang *et al*. 2021) and may have been indicative of stable declarative knowledge of the CS-US pairing even after extinction. Importantly, the fMRI findings were largely consistent with past research assessing an extinguished versus unextinguished CS+ during extinction recall (Milad *et al*. 2007, Fullana *et al*. 2018).

Most notably, during the Visual Extinction Recall phase the vmPFC displayed greater activity for the CS+Ei than the CS+U. Moreover, when examining this effect by phase, vmPFC activity was not different between the CS+U and CS+Ei during acquisition. However, after imagined extinction, vmPFC activity was significantly greater for the CS+Ei compared to the CS+U during the Visual Extinction Recall phase. This finding of vmPFC activity during extinction recall is similar to studies of perceptual extinction that use a within-subject designs to compare the CS+E to the CS+U (Milad *et al*. 2007, Fullana *et al*. 2018). Additionally, although we observed no univariate vmPFC activity during extinction learning, the differential vmPFC findings for the CS+Ei versus CS+U during extinction recall possibly suggest that imagined extinction can affect similar regions to perceptual extinction. Following these vmPFC findings, mixed evidence of extinction recall was found in the bilateral dACC and L-AIC. Throughout this phase, both regions were more active during the CS+Ei than the CS-. One possible interpretation is that the CS+Ei activation reflected fear recall from the Acquisition phase, rather than fear extinction. Alternatively, these regions may serve a different function during extinction as compared to acquisition (Fullana *et al*. 2018). Consistent with this alternative, previous meta-analytic findings revealed that activation in these same regions was associated with successful extinction learning and recall (Fullana *et al*. 2018). Moreover, for the dACC and L-AIC, the CS+U was not significantly different from the CS−, which could be interpreted as a lack of persistent fear for the CS+U. Yet, convergent results from other measures of fear indicated greater responding for CS+U than CS− in SCR, self-report, and vmPFC activity, which indicates that fear did persist for the CS+U. Finally, during extinction recall, while only the CS+Ei was greater than the CS− in the L-AIC, both the CS+Ei and CS+U were greater than the CS− in the R-AIC, which corroborates meta-analytic findings of extinction recall (Fullana *et al*. 2018).

The vmPFC findings of the present study appear consistent with previous research suggesting that the vmPFC plays an important inhibitory role during extinction recall and emotion regulation (Schiller *et al*. 2013). Overlapping animal (Milad *et al*. 2004, Quirk and Beer 2006) and human (Phelps *et al*. 2004, Milad *et al*. 2007) research have both observed that the vmPFC may be crucial in the retention and expression of an extinction memory. Structural research has implicated the cortical thickness of the vmPFC in down-regulation of fear conditioning (Milad *et al*. 2004). Moreover, hypoactivity in this region, due to lesions (Nieuwenhuis and Takashima 2011) or sleep loss (Ben Simon *et al*. 2020), has also been shown to affect one’s ability to inhibit a fear response. Furthermore, increased vmPFC activity may work to attenuate limbic regions, such as the amygdala, which will in turn lower arousal (Phelps *et al*. 2004, Milad *et al*. 2007). Meta-analytic findings of brain imaging results in humans may be inconclusive regarding amygdala activity during fear extinction (Fullana *et al*. 2018). Some recent research finds that a temporal analysis of early trials is more likely to involve the amygdala (Wen *et al*. 2022, Radua *et al*. 2025), though the current study did not find significant amygdala activity using this approach. Rather, results from this study demonstrated significant vmPFC to amygdala connectivity for the CS+U in comparison to the CS+Ei during the Visual Extinction Recall phase. Notably, this finding appears inconsistent with connectivity studies in rodents that find stronger connectivity for the CS+E compared to the CS+U during extinction recall (Sotres-Bayon and Quirk 2010). Additionally, results from human studies suggest that increased connectivity between the vmPFC and amygdala may indicate greater regulation as the increased vmPFC activity inhibits the amygdala (Milad *et al*. 2007). Thus, one possible inference is that the CS+U during visual extinction recall produced greater regulation in comparison to the CS+Ei. However, human neuroimaging research has also found that differences in age, anxiety, and threat related attention can produce opposite trends regarding amygdala-vmPFC connectivity (Gold *et al*. 2016). Further, the vmPFC has been highlighted as a multifaceted brain region that plays several roles in psychological function (Hiser and Koenigs 2018). Speculatively, another possible explanation of the current results is that the heightened connectivity during the CS+U reflects the vmPFC actively creating an extinction memory. Said another way, the CS+U may have shown stronger connectivity as the vmPFC to amygdala inhibitory connections were being expressed in response to the acquisition of the extinction memory; in contrast, this process may have already been completed for the CS+Ei. Another possible factor is that the present study did not provide a consolidation period between the extinction learning and the extinction recall phases, as found in similar research (Milad *et al*. 2007). Instead, our procedure was a single day design consistent with the only other study to examine fear extinction learning by imagery in fMRI (Reddan *et al*. 2018). A final consideration for these connectivity results is the extinction memory was created via mental imagery, a perhaps entirely human phenomenon. In this way, human imagery could have contributed to this seemingly inconsistent finding with the rodent and perceptual extinction literature. Taken together, it is presently unclear how immediate extinction, or mental imagery may have contributed to the connectivity results. Future research could assess how these factors may affect the connectivity of brain structures.

A final consideration ought to be made when interpreting these results regarding our ROI approach and our choice of functional localizer. While the localizer used in this study prioritized generalizable affective regions, a more specific localizer for differential fear conditioning may have produced a more sensitive fear circuit.

The current study found that imagery of a previously feared stimulus devoid of consequence displayed extinction qualities akin to those found in perceptual extinction studies (Milad *et al*. 2007, Pace-Schott *et al*. 2013). These findings are also consistent with the burgeoning number of experimental psychophysiological studies comparing imagined extinction to perceptual extinction (Agren *et al*. 2017, Grégoire and Greening 2019, Jiang *et al*. 2021, Hoppe *et al*. 2022, Mitra and Asthana 2024). However, future investigations may require more imagined extinction trials or longer extinction windows as imagery may yield a weaker extinction outcome than perceptual extinction. Nevertheless, along with Reddan *et al*. (2018), the current study further demonstrated the ability of imagined extinction recall to engage aspects of the vmPFC which appear involved in extinction learning and recall. Such findings may explain why imagined extinction can be an effective clinical strategy for the regulation of fear or anxiety (Holmes and Mathews 2010, Mitra and Asthana 2024).

## Supplementary Material

nsag031_Supplementary_Data

## Data Availability

The data underlying this article are available in “Mental Imagery-based Fear Extinction,” at doi: 10.18112/openneuro.ds004407.v1.0.0.
