# Supplementary material for: Ventromedial prefrontal cortex activity during extinction recall suggests successful extinction learning via mental imagery
Source: Soc Cogn Affect Neurosci. 2026 May 7;21(1):nsag031. doi: 10.1093/scan/nsag031 (PMC13215085; doi:10.1093/scan/nsag031)
Supplement: nsag031_Supplementary_Data [file nsag031_supplementary_data.docx]

**Supplemental Materials**

1. Exploratory time analysis by phase for SCR:

*1.1 Acquisition Phase:*

As an exploratory analysis, mean SCR was analyzed for the first and second halves of the phase with a 3 (CS type: CS+U vs. CS+Ei vs. and CS-) x 2 (Time: First vs. Second) ANOVA. There was no main effect of Time, *F*(1, 27) = 0.02, p = .967, *η_p_*^2^ < .01. As well, there was no significant interaction, *F*(2, 54) = 3.34, p = .056, *η_p_*^2^ = .10. However, paired sample t-tests demonstrated that the CS+U was not significantly greater than the CS- during the first half of the phase, *t*(27) = 1.87, *p* = .073, *d* = 0.34, but gained significance during the second half of the phase, *t*(27) = 3.84, *p* < .001, *d* = 0.73. Alternatively, the CS+Ei was both significantly greater than the CS- during the first half of the phase, *t*(27) = 2.57, *p* = .008, *d* = 0.49, and the second half of the phase, *t*(27) = 2.33, *p* = .028, *d* = 0.44.

*1.2 Imagery Extinction Learning Phase:*

As an exploratory analysis, mean SCR was separated into early (first half of trials) and late (second half of trials) responses during the Imagery Extinction Learning phase. A 2x2 repeated measures ANOVA with CS type (i.e., CS+Ei and CS-) and time (i.e., early and late) as the within-subject factors revealed a significant main effect for CS type, *F*(1, 26) = 5.52, *p* = .027, *η_p_*^2^ = .18. There was no significant main effect of Time, *F*(1, 26) = 3.54, *p* = .071, *η_p_*^2^ = .12, and no interaction, *F*(1, 26) = 0.35, *p* = .558, *η_p_*^2^ = .01. Paired sample t-tests indicated that the CS+Ei was greater than the CS-, *t*(26) = 2.41, *p* = .023, *d* = 0.47, during the early portion of the phase. This differential then extinguished during the late portion of the phase, *t*(26) = 1.71, *p* = .100, *d* = 0.33.

*1.3 Visual Extinction Recall Phase:*

Mean SCR were again separated into early (first half of trials) and late (second half of trials) visual extinction recall responses. A 3x2 repeated measures ANOVA with CS type (i.e., CS+U, CS+Ei, and CS-) and Time (i.e., early and late) as the within-subject factors reported significant main effects of CS type, *F*(2, 52) = 4.30, *p* = .019, *η_p_*^2^ = .14, and Time, *F*(1, 26) = 6.17, *p* = .020, *η_p_*^2^ = .19, with greater mean SCR in the early portion of the phase compared to the late portion of the phase. No significant interaction was found, *F*(2, 52) = 2.54, *p* = .088, *η_p_*^2^ = .09.

*2. Exploratory analysis of the amygdala by time:*

During acquisition, there was no main effect of CS Type in the L-amygdala, *F*(2, 54) = 0.34, *p* = .716, *η_p_*^2^ = .01, or R-amygdala, *F*(2, 54) = 0.57, *p* = .568, *η_p_*^2^ = .02; no main effect of Time in the L-amygdala, *F*(1, 27) = 0.69, *p* = .412, *η_p_*^2^ = .03, or R-amygdala, *F*(1, 27) = 0.06, *p* = .814, *η_p_*^2^ < .01; nor a significant interaction in the L-amygdala, *F*(2, 54) = 2.66, *p* = .079, *η_p_*^2^ = .09, or R-amygdala, *F*(2, 54) = 0.313, *p* = .732, *η_p_*^2^ = .01.

Similar to acquisition, during extinction learning, there was no main effect of CS Type in the L-amygdala, *F*(1, 27) < 0.01, *p* = .983, *η_p_*^2^ < .01, or R-amygdala, *F*(1, 27) = 0.08, *p* = .785, *η_p_*^2^ < .01; no main effect of Time in the L-amygdala, *F*(1, 27) = 0.26, *p* = .613, *η_p_*^2^ = .01, or R-amygdala, *F*(1, 27) < 0.01, *p* = .983, *η_p_*^2^ < .01; nor a significant interaction in the L-amygdala, *F*(1, 27) = 0.83, *p* = .371, *η_p_*^2^ = .03, or R-amygdala, *F*(1, 27) = 1.28, *p* = .268, *η_p_*^2^ = .05.

Finally, similar to both previous phases, during extinction recall, there was no main effect of CS Type in the L-amygdala, *F*(2, 54) = 2.12, *p* = .130, *η_p_*^2^ = .07, or R-amygdala, *F*(2, 54) = 2.79, *p* = .070, *η_p_*^2^ = .09; no significant main effect of Time in the L-amygdala, *F*(1, 27) = 3.28, *p* = .081, *η_p_*^2^ = .11, or R-amygdala, *F*(1, 27) = 1.53, *p* = .226, *η_p_*^2^ = .05; nor a significant interaction in the L-amygdala, *F*(2, 54) = 0.26, *p* = .775, *η_p_*^2^ = .01 or R-amygdala, *F*(2, 54) = 0.35, *p* = .706, *η_p_*^2^ = .01.
